# Association between a novel inflammation-lipid composite marker CRP/HDL and erectile dysfunction: evidence from a large national cross-sectional study

**DOI:** 10.3389/fendo.2024.1492836

**Published:** 2024-11-20

**Authors:** Yangyang Mei, Yiming Chen, Bo Zhang, Wei Xia, Naiyuan Shao, Xingliang Feng

**Affiliations:** ^1^ Department of Urology, Jiangyin People’s Hospital, the Jiangyin Clinical College of Xuzhou Medical University, Jiangyin, Jiangsu, China; ^2^ Department of Urology, The Third Affiliated Hospital of Soochow University, Changzhou, Jiangsu, China; ^3^ Department of Urology, The First People’s Hospital of Changzhou, Changzhou, Jiangsu, China; ^4^ Department of Neurosurgery, The Third Affiliated Hospital of Soochow University, Changzhou, Jiangsu, China; ^5^ Department of Neurosurgery, The First People’s Hospital of Changzhou, Changzhou, Jiangsu, China

**Keywords:** NHANES, erectile dysfunction, inflammation, high-density lipoprotein, cross-sectional study

## Abstract

**Background:**

Erectile dysfunction (ED) is one of the most common male sexual disorders, closely associated with both inflammation and lipid dysregulation. Recently, a novel inflammation-lipid composite marker, CRP/HDL, has been proposed to integrate the impact of both pathways on health, yet its relationship with ED remains unexplored. Therefore, our study aimed to investigate the potential association between the CRP/HDL ratio and ED.

**Methods:**

We utilized data from the NHANES database, known for its comprehensive and high-quality information. A total of 3,633 eligible participants from the 2001-2004 cycles were included. ED was assessed using a single-item questionnaire, while CRP and HDL were measured from blood samples. Multivariable regression analyses were performed to evaluate the association between CRP/HDL and ED after adjusting for potential confounders. Additionally, subgroup and sensitivity analyses were conducted to test the robustness of the results, and the linear trend between CRP/HDL and ED was visualized through smooth curve fitting.

**Results:**

Among the 3,633 participants, 1,027 had a history of ED. The CRP/HDL ratio was significantly higher in participants with ED compared to those without (10.53 ± 0.69 vs. 7.43 ± 0.35, P<0.001). In the regression analysis, a higher continuous CRP/HDL ratio was significantly associated with increased ED risk even after full adjustment (OR: 1.17, 95% CI: 1.05, 1.30; P = 0.01). Compared to Q1 of the CRP/HDL ratio, participants in Q2, Q3, and Q4 had progressively higher ED risks: Q2 (OR: 1.40, 95% CI: 1.01, 1.95; P = 0.05), Q3 (OR: 1.58, 95% CI: 1.10, 2.27; P = 0.02), and Q4 (OR: 1.85, 95% CI: 1.31, 2.60; P = 0.005), showing a clear linear trend. Subgroup analyses indicated consistent results across various populations with no significant interactions, and sensitivity analysis revealed that the CRP/HDL ratio also increased the risk of severe ED (OR: 1.14, 95% CI: 1.03, 1.26; P = 0.02).

**Conclusion:**

This is the first study to establish a significant positive association between an elevated CRP/HDL ratio and ED risk, suggesting its potential role in screening for ED risk and guiding timely interventions. However, further large-scale studies are needed to confirm our findings and explore the underlying mechanisms.

## 1 Introduction

Erectile dysfunction (ED) is one of the most prevalent male sexual disorders, characterized by the inability to achieve or maintain sufficient penile rigidity for satisfactory sexual performance (1). Historically, ED was widely regarded as a psychological condition (2). However, advancements in diagnostic techniques have revealed that more than 80% of cases are attributable to organic factors (3), leading to the classification of ED into psychogenic, organic, and mixed types (4). Among organic causes, vascular etiology is recognized as the predominant factor, given the highly vascularized nature of penile tissue (5, 6). It is estimated that up to 70% of organic ED cases have a vascular origin (7). Alarmingly, recent projections estimate that by 2025, the global prevalence of ED will rise to 322 million cases, signaling a significant public health concern (8). Consequently, identifying reliable predictive markers for early detection of ED risk and enabling timely intervention has become increasingly important.

In addition to being a growing public health concern, ED also serves as an early clinical indicator of systemic vascular disease and represents an independent risk factor for cardiovascular events (9–11). Research has demonstrated a strong association between ED and the presence as well as the severity of asymptomatic atherosclerosis (12). Moreover, low-grade subclinical inflammation contributes to endothelial dysfunction, playing a role throughout all stages of the atherosclerotic process (13, 14). Consequently, the risk factors for both ED and cardiovascular disease (CVD) are shared, including obesity, hypertension, metabolic syndrome, and diabetes mellitus (DM), all of which are linked to vascular inflammation that subsequently impairs endothelial function (10, 14, 15). Furthermore, current inflammatory markers, such as C-reactive protein (CRP), systemic immune-inflammation index (SII), and neutrophil-to-lymphocyte ratio (NLR), have demonstrated strong associations with ED (16, 17). A meta-analysis reported significantly elevated CRP levels in patients with ED compared to healthy controls (SMD (95% CI) = 0.97 (0.55, 1.40), p < 0.001) (18). Additionally, lipid metabolism disorder is also a critical risk factor for ED, contributing to localized atherosclerosis through inflammatory pathways (19). Similarly, lipid metabolism markers, including total cholesterol (TC), triglycerides (TG), high-density lipoprotein (HDL), and related ratios, have been increasingly recognized as relevant to ED risk (20, 21). One study, which used penile peak systolic velocity (PSV) to assess penile arterial blood flow, found a significant positive correlation between HDL levels and PSV (r=0.471, P<0.01) (22). These results suggested that both inflammation and lipid metabolism play crucial roles in the development of ED by affecting penile arterial function (11, 23, 24). Despite these findings, most existing studies on ED risk prediction have focused on either inflammatory or lipid markers individually, which provides an incomplete assessment (25, 26). Research also suggests that inflammation and lipid metabolism are interrelated processes (27). Therefore, a composite marker integrating both aspects may offer a more comprehensive evaluation of ED risk.

Recently, a large-scale national study evaluated the novel CRP/HDL ratio and demonstrated its predictive value for CVD, providing a solid theoretical basis (28). Moreover, the close relationship between CVD and ED, grounded in shared endothelial dysfunction, has been substantiated by multiple studies (29). Another key consideration is that both CRP and HDL are widely used, cost-effective clinical markers. Therefore, we sought to investigate whether this composite marker is also linked to ED and could be used to assess ED risk. Utilizing the extensive and high-quality data from the NHANES database, we aim to explore the association between this inflammation-lipid composite marker, CRP/HDL, and ED. We hypothesize that an elevated CRP/HDL ratio is positively correlated with ED risk and propose that this marker could be clinically useful in assessing ED risk and guiding early interventions to protect men’s sexual health.

## 2 Materials and methods

### 2.1 Study design and population

To assess the nutritional and health status of the U.S. population and provide data-driven guidance for public health policy, the National Center for Health Statistics (NCHS), under the CDC, implemented a continuous, nationwide survey targeting U.S. children and adults. This program, known as NHANES, gathers diverse data through a combination of household interviews and physical examinations conducted at mobile examination centers (MEC). The collected information spans demographic characteristics, dietary habits, physical measurements, laboratory tests, and self-reported questionnaires. To ensure a representative sample of the non-institutionalized U.S. population, NHANES employs a complex, multistage, stratified sampling design. All human research within the study received prior approval from the NCHS Ethics Review Board (Approval No. Protocol #98-12), and written informed consent was obtained from all participants in compliance with ethical guidelines.

**Figure 1:**
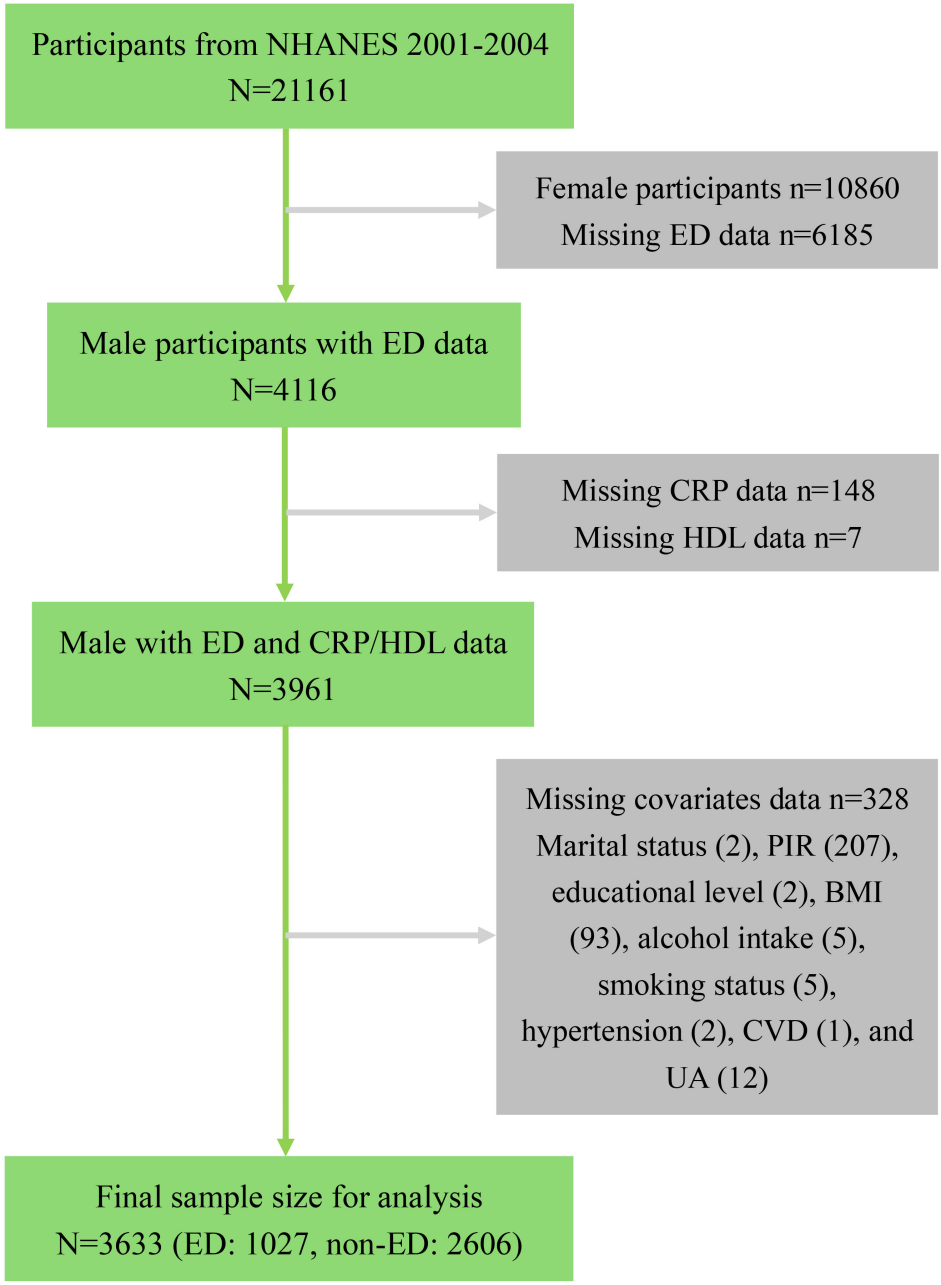
Flowchart of sample selection from NHANES 2001-2004. NHANES, National Health and Nutrition Examination Survey; ED, Erectile dysfunction; CRP, C-reactive protein; HDL, High-density lipoprotein; PIR, Poverty income ratio; BMI, Body mass index; UA, Uric acid; CVD, Cardiovascular disease.

For this analysis, we focused on participants from the 2001-2004 NHANES cycles, as the relevant sexual function questionnaire was administered only during this period, initially yielding a potential sample of 21,161 participants. We first excluded 10,860 female participants. Subsequently, 6,185 participants were excluded due to missing ED data, including those under 20 years old or those who refused to answer. We then removed participants with missing data necessary for calculating the CRP/HDL ratio, specifically 148 lacking CRP data and 7 missing HDL measurements. Additionally, to enhance analytical precision, we excluded 328 participants with incomplete covariate data, with specific exclusion criteria detailed in **Figure 1**. After applying these criteria, a total of 3,623 eligible participants remained, comprising 1,027 with a history of ED and 2,606 without.

### 2.2 Exposure variable assessment

The exposure variable in this study is derived from two biomarkers: CRP and HDL. All blood samples were collected after participants fasted for at least 8.5 hours. Sample preparation, storage, and transport adhered to standardized protocols, and subsequent analyses were conducted in certified laboratories under the supervision of NCHS. NHANES 2001-2004 CRP levels, rather than high-sensitivity CRP, were measured using latex-enhanced nephelometry on a Behring Nephelometer, while HDL levels were determined through direct precipitation or immunoassays. Additionally, the detection limits for both CRP and HDL were set at 0. More detailed information can be found on the NHANES official website. For regression analyses, the CRP/HDL ratio was log-transformed to enhance interpretability of the odds ratios (ORs). Higher CRP/HDL ratios indicate greater vascular inflammation and reduced protective HDL levels. These biomarkers are routinely assessed in clinical settings, offering an easily accessible, cost-effective, and objective measure. Detailed laboratory procedures are available on the NHANES website.

### 2.3 Outcome variable evaluation

While most studies assess erectile function using the International Index of Erectile Function-5 (IIEF-5) questionnaire, NHANES 2001-2004 employed a single-item question derived from the Massachusetts Male Aging Study (MMAS) (30, 31). This question aligns with items 2, 3, and 5 from the IIEF-5 and has been validated across multiple studies related to ED using NHANES data (32, 33). Eligible participants were asked to evaluate their ability to achieve and maintain an erection sufficient for satisfactory sexual intercourse. The responses included four options: “always or almost always able,” “usually able,” “sometimes able,” or “never able.” Participants who responded “always or almost always able” or “usually able” were classified as having normal erectile function, while all other responses indicated the presence of ED. Based on existing literature, severe ED was further defined by selecting participants who responded “never able” to maintain an erection during sexual intercourse (34).

### 2.4 Definition of covariates

Based on existing literature, a number of potential covariates that may influence the relationship between CRP/HDL and ED were considered, including age, race/ethnicity, marital status, education level, poverty income ratio (PIR), body mass index (BMI), smoking status, alcohol consumption, hypertension, DM, CVD, and hyperlipidemia. Additionally, serum uric acid (UA) and total cholesterol (TC) were also included. Age was categorized as <60 years and ≥60 years. Race/ethnicity was classified as Mexican American, Non-Hispanic White, Non-Hispanic Black, Other Hispanic, and other races. Marital status was divided into “Married or living with a partner” and “Living alone.” Education level was grouped as “Below high school,” “High school,” and “Above high school.” PIR was stratified into three categories: PIR ≤ 1.3, 1.3<PIR ≤ 3.5, and PIR>3.5. These demographic factors were obtained through standardized questionnaires. BMI was calculated as weight in kilograms divided by height in meters squared (kg/m²) and was measured during the MEC visit. Smoking status was determined by asking participants whether they had smoked at least 100 cigarettes in their lifetime and their current smoking status. Participants were classified as “never” (neither criteria met), “current” (both criteria met), or “former” (remaining participants). Alcohol consumption was categorized based on whether participants had consumed more than 12 drinks in the past year. The definitions for comorbidities were consistent with established guidelines. Hypertension was defined by a history of diagnosis, current antihypertensive medication use, or an average blood pressure reading of ≥140/90 mmHg. Hyperglycemia was defined by a self-reported diabetes diagnosis, use of antidiabetic medications or insulin, a plasma glucose level ≥200 mg/dL at 2 hours post-oral glucose tolerance test (OGTT), HbA1c ≥6.5%, or a fasting glucose level ≥126 mg/dL. Hyperlipidemia was defined by either a history of diagnosis, use of lipid-lowering medications, or a total cholesterol value ≥240 mg/dL. CVD was identified based on a history of congestive heart failure, coronary heart disease, angina, or heart attack. Further details on the covariates can be accessed from the NHANES website (www.cdc.gov/nchs/nhanes/).

### 2.5 Statistical analysis

Considering the complex sampling design and representative nature of NHANES, all statistical analyses accounted for the survey design and applied appropriate weights, specifically the 2-year MEC sample weights divided by 2. For the initial descriptive analysis, continuous variables were presented as weighted means ± standard errors, with group comparisons conducted using weighted linear regression. Categorical variables were expressed as weighted percentages, and group differences were assessed using weighted chi-square tests. The association between the CRP/HDL ratio and ED prevalence was evaluated using both univariate and multivariate logistic regression models across three levels of adjustment. Model 1 included only the exposure variable without additional covariates (unadjusted model). Model 2 adjusted for demographic factors, including age, race/ethnicity, marital status, education level, and PIR (minimally adjusted model). Model 3 further adjusted for BMI, TC, UA, smoking status, alcohol consumption, and histories of hypertension, DM, CVD, and hyperlipidemia (fully adjusted model). To explore potential linear trends, the continuous CRP/HDL ratio was categorized into quartiles and included in the weighted multivariate regression models. All regression results were reported as odds ratios (OR) with 95% confidence intervals (CI). Additionally, to visually demonstrate the relationship between the CRP/HDL ratio and ED, smooth curve fitting and generalized additive models were employed.

To assess the stability of the CRP/HDL-ED relationship across different population characteristics, subgroup analyses were conducted, adjusting for all covariates in Model 3 except the stratified variable itself. Both continuous and quartile-categorized CRP/HDL ratios were included in the models for subgroup analyses. Sensitivity analyses were performed to confirm the robustness of the findings by redefining the outcome as severe ED, where only participants who responded “never able” to maintain an erection during sexual intercourse were considered. Statistical significance was defined as a two-sided p-value < 0.05. EmpowerStats (http://www.empowerstats.com, X&Y Solutions, Inc.) and R statistical software (http://www.R-project.org; The R Foundation) were used for all analyses.

## 3 Results

### 3.1 Baseline characteristics of the study participants

The baseline characteristics of the study population are presented in **Table 1**. A total of 3,633 participants were included in the final analysis, of whom 1,027 had a history of ED, representing a prevalence of 28.27%. Significant differences were observed between the ED and non-ED groups in most demographic and clinical characteristics, except for several hematological measures and race. Specifically, participants with ED were older, had higher BMI, lower education levels, and a greater likelihood of comorbid conditions such as hypertension, DM, hyperlipidemia, and CVD. Notably, the CRP/HDL ratio was substantially higher in participants with ED compared to those without ED (10.53 ± 0.69 vs. 7.43 ± 0.35, P<0.001), underscoring the potential link between increased inflammatory burden, reduced protective lipid levels, and ED risk.

**Table 1:** Baseline characteristics of the study participants from NHANES 2001-2004, weighted.

Characteristics	Total participants	History of ED		P value
		No	Yes	
Number, n	3633	2606	1027	
Age, year	45.04 ± 0.37	41.29 ± 0.30	61.02 ± 0.54	< 0.0001
BMI, kg/m^2^	28.12 ± 0.11	27.89 ± 0.12	29.08 ± 0.27	< 0.001
TC, mg/dL	201.52 ± 1.12	202.00 ± 1.13	199.47 ± 2.27	0.27
UA, mg/dL	6.11 ± 0.03	6.10 ± 0.03	6.16 ± 0.06	0.38
CRP, mg/dL	0.34 ± 0.01	0.31 ± 0.01	0.44 ± 0.03	< 0.0001
HDL, mg/dL	47.07 ± 0.34	47.15 ± 0.36	46.76 ± 0.47	0.42
CRP/HDL*1000	8.02 ± 0.33	7.43 ± 0.35	10.53 ± 0.69	< 0.001
Log (CRP/HDL*1000)	1.23 ± 0.03	1.14 ± 0.03	1.60 ± 0.04	< 0.0001
Age group, %				< 0.0001
<60y	63.54	73.92	19.48	
≥60y	36.46	26.08	80.52	
Body mass index (kg/m^2^), %				< 0.001
<25	29.22	30.52	23.70	
25-30	41.29	41.46	40.56	
>30	29.50	28.02	35.74	
Race, %				0.10
Mexican American	7.86	8.09	6.88	
Non-Hispanic White	74.55	73.96	77.05	
Non-Hispanic Black	9.35	9.64	8.09	
Other Hispanic	4.35	4.10	5.41	
Other races	3.90	4.21	2.56	
Educational level, %				< 0.0001
Below high school	16.61	13.73	28.84	
High school	27.06	27.89	23.54	
Above high school	56.33	58.38	47.62	
Marital status, %				< 0.0001
Married or living with a partner	29.46	31.13	22.38	
Living alone	70.54	68.87	77.62	
PIR, %				< 0.0001
PIR ≤ 1.3	16.18	15.66	18.39	
1.3<PIR ≤ 3.5	35.61	34.15	41.84	
PIR>3.5	48.21	50.19	39.77	
Alcohol intake, %				< 0.0001
No	23.41	20.36	36.37	
Yes	76.59	79.64	63.63	
Smoking, %				< 0.0001
Never	42.78	45.57	30.92	
Former	29.09	25.06	46.22	
Current	28.13	29.37	22.86	
History of DM, %				< 0.0001
No	89.79	94.03	71.81	
Yes	10.21	5.97	28.19	
History of CVD, %				< 0.0001
No	91.05	94.84	74.91	
Yes	8.95	5.16	25.09	
History of hypertension, %				< 0.0001
No	65.52	71.13	41.69	
Yes	34.48	28.87	58.31	
History of hyperlipidemia				< 0.0001
No	27.59	29.49	19.52	
Yes	72.41	70.51	80.48	

ED, Erectile dysfunction; BMI, Body mass index; TC, Total cholesterol; UA, Uric acid; CRP, C-reactive protein; HDL, High-density lipoprotein; PIR, Poverty income ratio; DM, Diabetes mellitus; CVD, Cardiovascular disease.

Continuous variables are expressed as weighted means ± standard errors and compared using weighted linear regression. Categorical variables are presented as weighted percentages and 95% confidence intervals, with group comparisons performed using weighted chi-square tests. All p-values are two-sided, with statistical significance defined as P < 0.05.

### 3.2 Association between CRP/HDL ratio and ED: findings from regression analysis

**Figure 2:**
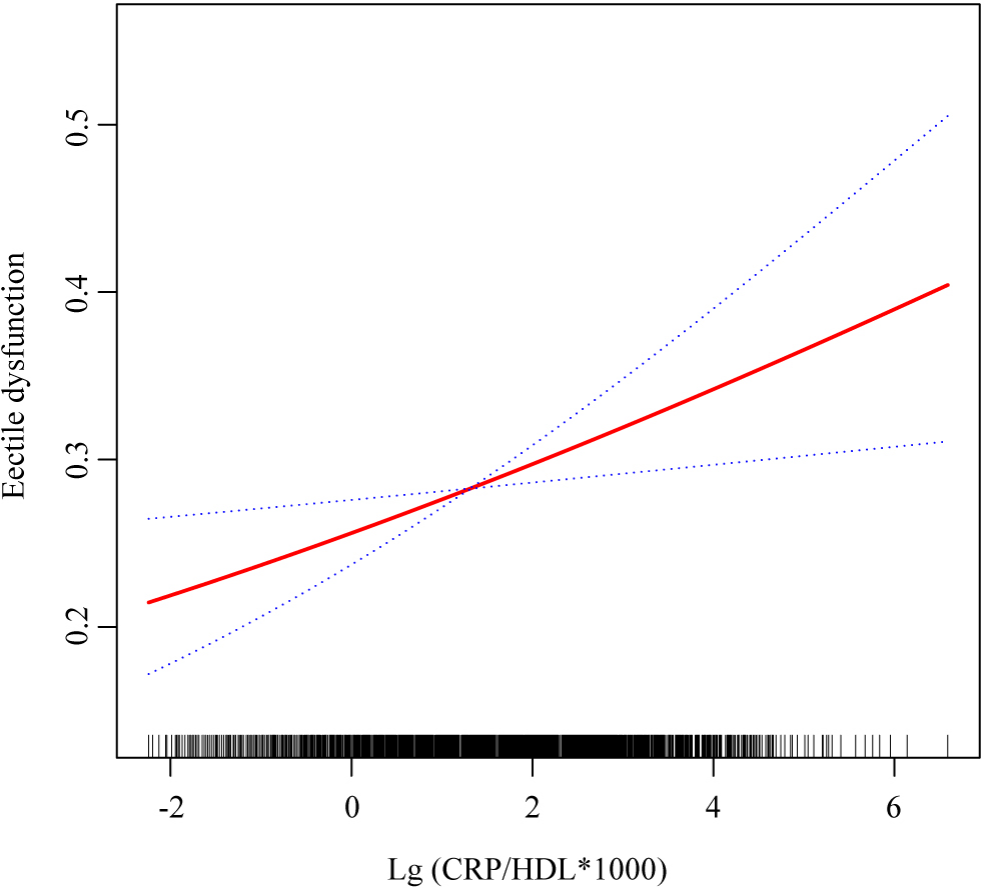
Smooth curve fitting showing the relationship between CRP/HDL ratio and ED. This figure visualizes the association between the log-transformed CRP/HDL ratio and the probability of ED using smooth curve fitting. The red solid line represents the fitted association, while the blue dashed lines indicate the 95% confidence intervals. The analysis was adjusted for all covariates included in Model 3: age, race/ethnicity, education level, PIR, marital status, BMI, TC, UA, alcohol intake, smoking status, hypertension, diabetes, CVD, and hyperlipidemia. CRP, C-reactive protein; HDL, High-density lipoprotein; ED, Erectile dysfunction; PIR, Poverty income ratio; BMI, Body mass index; TC, Total cholesterol; UA, Uric acid; CVD, Cardiovascular disease.

Multivariable regression analyses were conducted to explore the relationship between the CRP/HDL ratio and ED prevalence, with results presented in **Table 2**. To enhance the interpretability of ORs, the exposure variable was log-transformed when analyzed as a continuous variable. The findings indicate that across all models, the CRP/HDL ratio was positively and significantly associated with the prevalence of ED. The ORs for the association were as follows: Model 1 (unadjusted) 1.34 (95% CI: 1.25, 1.43; P < 0.0001), Model 2 (minimally adjusted) 1.21 (95% CI: 1.10, 1.32; P < 0.001), and Model 3 (fully adjusted) 1.17 (95% CI: 1.05, 1.30; P = 0.01). Next, the CRP/HDL ratio was categorized into quartiles, with Q1 serving as the reference. Across all models, participants in Q2, Q3, and Q4 exhibited significantly increased risks of ED. In the fully adjusted Model 3, the ORs for ED were as follows: Q2, 1.40 (95% CI: 1.01, 1.95; P = 0.05); Q3, 1.58 (95% CI: 1.10, 2.27; P = 0.02); and Q4, 1.85 (95% CI: 1.31, 2.60; P = 0.005). A trend test confirmed a statistically significant dose-response relationship (p for trend < 0.05). Lastly, we visually demonstrated the linear relationship between the CRP/HDL ratio and ED prevalence using smooth curve fitting (**Figure 2**). The results further corroborate the presence of a linear trend, reinforcing the potential role of the CRP/HDL ratio as a predictor of ED risk.

**Table 2:** Multivariable logistic regression analysis of the association between CRP/HDL ratio and ED, weighted.

Exposure	Model 1		Model 2		Model 3	
	OR (95%CI)	P value	OR (95%CI)	P value	OR (95%CI)	P value
Log (CRP/HDL*1000)	1.34(1.25,1.43)	<0.0001	1.21(1.10, 1.32)	<0.001	1.17(1.05, 1.30)	0.01
CRP/HDL*1000, quartiles						
Q1 (<1.556)	Reference		Reference		Reference	
Q2 (1.556-3.704)	1.81(1.42,2.31)	<0.0001	1.46(1.13, 1.89)	0.01	1.40(1.01, 1.95)	0.05
Q3 (3.704-8.444)	2.14(1.58,2.88)	<0.0001	1.65(1.19, 2.27)	0.005	1.58(1.10, 2.27)	0.02
Q4 (>8.444)	2.97(2.29,3.86)	<0.0001	2.05(1.52, 2.77)	<0.001	1.85(1.31, 2.60)	0.005
P for trend	<0.0001		<0.001		0.003	

CRP, C-reactive protein; HDL, High-density lipoprotein; PIR, Poverty income ratio; BMI, Body mass index; TC, Total cholesterol; UA, Uric acid; CVD, Cardiovascular disease; DM, Diabetes mellitus; ED, Erectile dysfunction; OR, Odds ratio; CI, Confidence interval.

The regression models evaluate the association between the CRP/HDL ratio and ED risk.

Model 1 includes only the CRP/HDL ratio without adjustment.

Model 2 adjusts for demographic factors (age, race/ethnicity, marital status, education level, and PIR).

Model 3 further adjusts for clinical factors (BMI, TC, UA, smoking status, alcohol consumption, hypertension, DM, CVD, and hyperlipidemia). The results are presented as ORs with 95% CIs, and P-values <0.05 are considered statistically significant. Trend tests across quartiles were conducted to assess the dose-response relationship.

### 3.3 Subgroup analysis: consistency of the CRP/HDL-ED association across diverse populations

**Figure 3:**
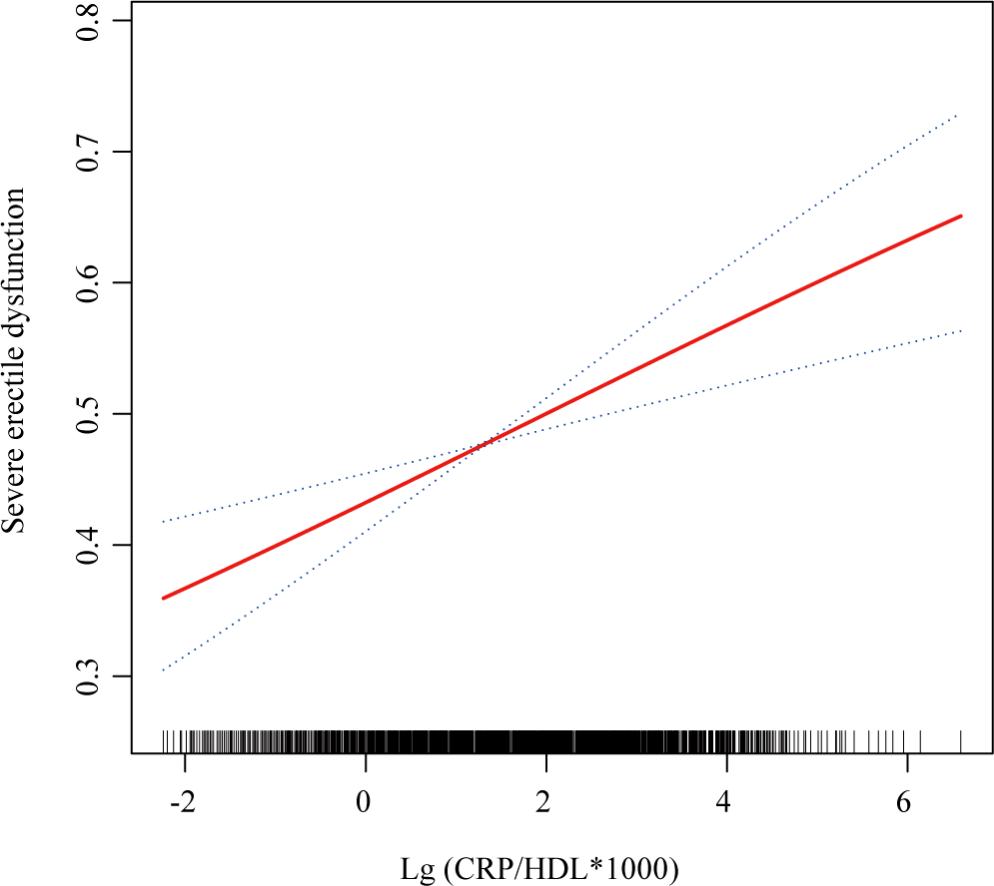
Subgroup analysis of the association between CRP/HDL ratio quartiles and ED, weighted. Subgroup analyses were analyzed across various population characteristics, with CRP/HDL analyzed as quartiles (Q1 as the reference). All analyses were adjusted for covariates included in Model 3: age, race/ethnicity, education level, PIR, marital status, BMI, TC, UA, alcohol intake, smoking status, hypertension, DM, CVD, and hyperlipidemia, excluding the stratified variable itself. ORs with 95%CI are presented for each subgroup. Trend tests across quartiles and interaction tests to assess heterogeneity between subgroups were also conducted, with statistical significance defined as a two-sided p-value < 0.05. CRP, C-reactive protein; HDL, High-density lipoprotein; BMI, Body mass index; PIR, Poverty income ratio; CVD, Cardiovascular disease; DM, Diabetes mellitus; ED, Erectile dysfunction; OR, Odds ratio; CI, Confidence interval.

Subgroup analyses were conducted to evaluate the stability of the CRP/HDL-ED relationship across key population characteristics, aligned with the findings from the regression analysis. First, when considering CRP/HDL as a continuous variable, as shown in **Figure 3**, the association between CRP/HDL and ED was more pronounced in older participants (≥60 years) and those without comorbidities. However, the P-values for interaction tests across all subgroups were greater than 0.05, indicating no significant heterogeneity between subgroups. Next, the subgroup analysis results using quartiles of CRP/HDL, with Q1 as the reference group, are presented in **Table 3**. The results remained consistent with those for the continuous variable, reinforcing the stability and reliability of the CRP/HDL-ED association. Importantly, no significant interactions were detected between subgroups, further supporting the robustness of the relationship.

**Table 3:** Subgroup analysis of the association between CRP/HDL ratio and ED across different population characteristics, weighted.

Subgroup	CRP/HDL*1000				P for trend	P for interaction
	Q1 (n=910)	Q2 (n=910)	Q3 (n=904)	Q4 (n=909)		
Age						0.77
<60y	Ref	1.25(0.59, 2.66)	1.21(0.62, 2.37)	1.69(0.81, 3.56)	0.18	
≥60y	Ref	1.45(0.95,2.21)	1.70(1.07,2.71)	1.93(1.33,2.80)	0.004	
BMI						0.08
<25 kg/m^2^	Ref	1.00(0.54, 1.86)	1.09(0.56, 2.13)	1.37(0.59,3.14)	0.41	
25-30 kg/m^2^	Ref	1.18(0.77, 1.81)	1.96(1.03, 3.73)	2.23(1.22, 4.06)	0.01	
>30 kg/m^2^	Ref	3.39(1.34, 8.55)	2.13(0.90, 5.05)	2.47(1.17, 5.20)	0.42	
Marital status						0.58
Married or living with a partner	Ref	1.12(0.53, 2.37)	1.26(0.77, 2.07)	1.82(1.00, 3.30)	0.04	
Living alone	Ref	1.55(1.07, 2.26)	1.76(1.09, 2.85)	1.92(1.22, 3.00)	0.01	
Smoking						0.47
Never	Ref	1.55(0.84, 2.86)	1.79(0.91, 3.53)	1.80(0.98,3.31)	0.05	
Former	Ref	1.59(1.01, 2.48)	2.08(1.19, 3.66)	2.48(1.56, 3.95)	0.002	
Current	Ref	0.97(0.44, 2.13)	0.86(0.40, 1.82)	1.23(0.47, 3.19)	0.59	
DM						0.27
No	Ref	1.30(0.90, 1.87)	1.65(1.12, 2.45)	1.99(1.33, 2.98)	0.002	
Yes	Ref	1.72(0.56, 5.23)	1.02(0.34, 3.07)	1.25(0.40, 3.96)	0.92	
CVD						0.31
No	Ref	1.37(1.00, 1.88)	1.51(1.08, 2.10)	1.92(1.38, 2.68)	0.002	
Yes	Ref	1.54(0.67, 3.59)	2.32(0.89, 6.08)	1.62(0.67, 3.88)	0.27	
Hyperlipidemia						0.37
No	Ref	1.29(0.62, 2.69)	1.03(0.47, 2.28)	2.59(1.24,5.38)	0.03	
Yes	Ref	1.44(0.97, 2.13)	1.71(1.05, 2.80)	1.78(1.19, 2.67)	0.01	

CRP, C-reactive protein; HDL, High-density lipoprotein; BMI, Body mass index; PIR, Poverty income ratio; TC, Total cholesterol; UA, Uric acid; CVD, Cardiovascular disease; DM, Diabetes mellitus; ED, Erectile dysfunction; OR, Odds ratio; CI, Confidence interval.

All subgroup analyses were adjusted for the following covariates included in Model 3: age, race/ethnicity, education level, PIR, marital status, BMI, TC, UA, alcohol intake, smoking status, hypertension, DM, CVD, and hyperlipidemia, excluding the stratified variable itself. The CRP/HDL ratio was analyzed as quartiles (Q1 as the reference). Trend tests were conducted across quartiles, and interaction tests assessed heterogeneity between subgroups. Statistical significance was defined as a two-sided P-value < 0.05.

### 3.4 Robustness check: sensitivity analysis on the relationship between CRP/HDL and severe ED

**Figure 4:**
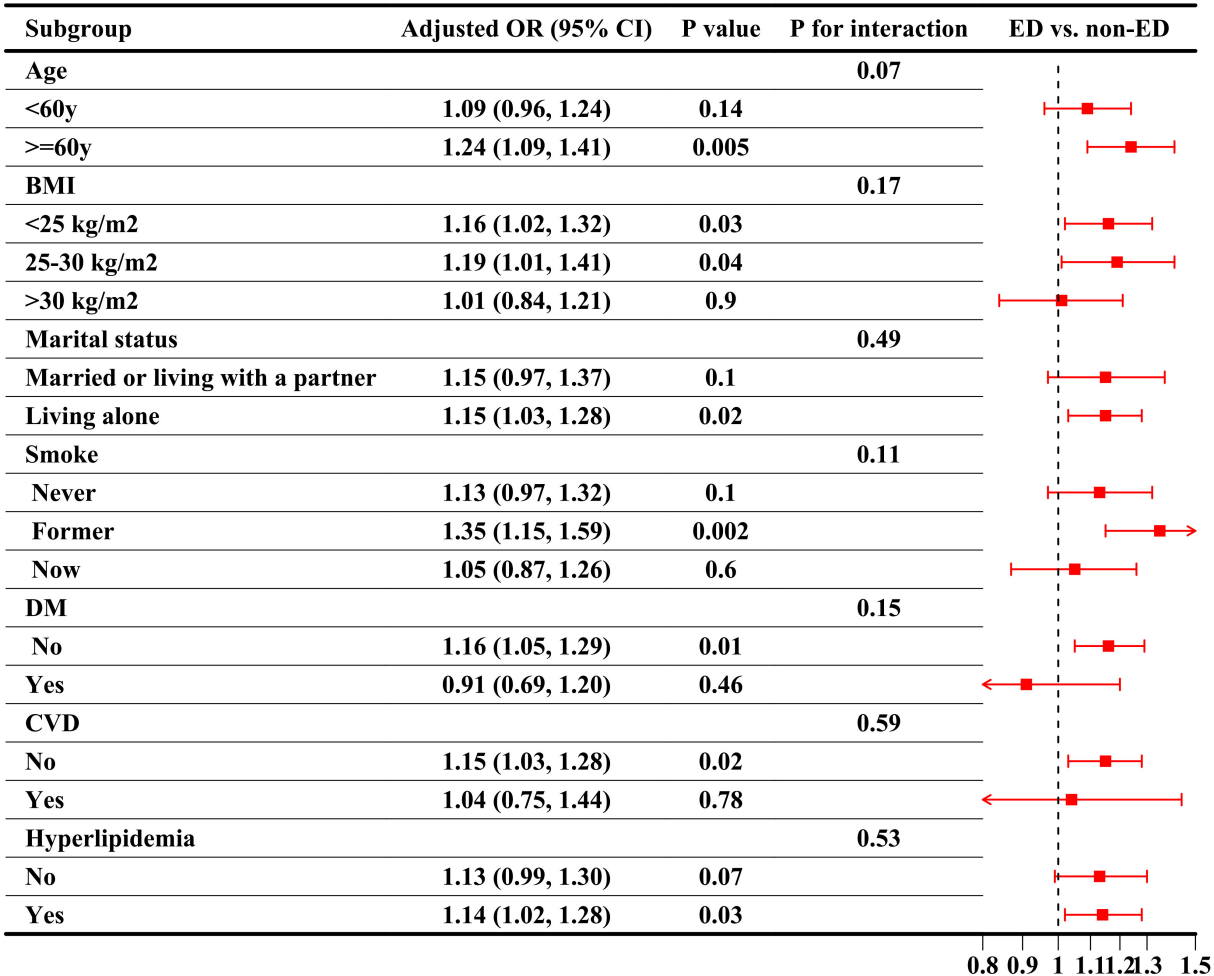
Smooth curve fitting showing the relationship between CRP/HDL ratio and severe ED. This figure visualizes the association between the log-transformed CRP/HDL ratio and the probability of severe ED using smooth curve fitting. The red solid line represents the fitted association, while the blue dashed lines indicate the 95%CI. The analysis was adjusted for all covariates included in Model 3: age, race/ethnicity, education level, PIR, marital status, BMI, TC, UA, alcohol intake, smoking status, hypertension, DM, CVD, and hyperlipidemia. CRP, C-reactive protein; HDL, High-density lipoprotein; ED, Erectile dysfunction; PIR, Poverty income ratio; BMI, Body mass index; TC, Total cholesterol; UA, Uric acid; DM, Diabetes mellitus; CVD, Cardiovascular disease; CI, Confidence interval.

**Figure 5:**
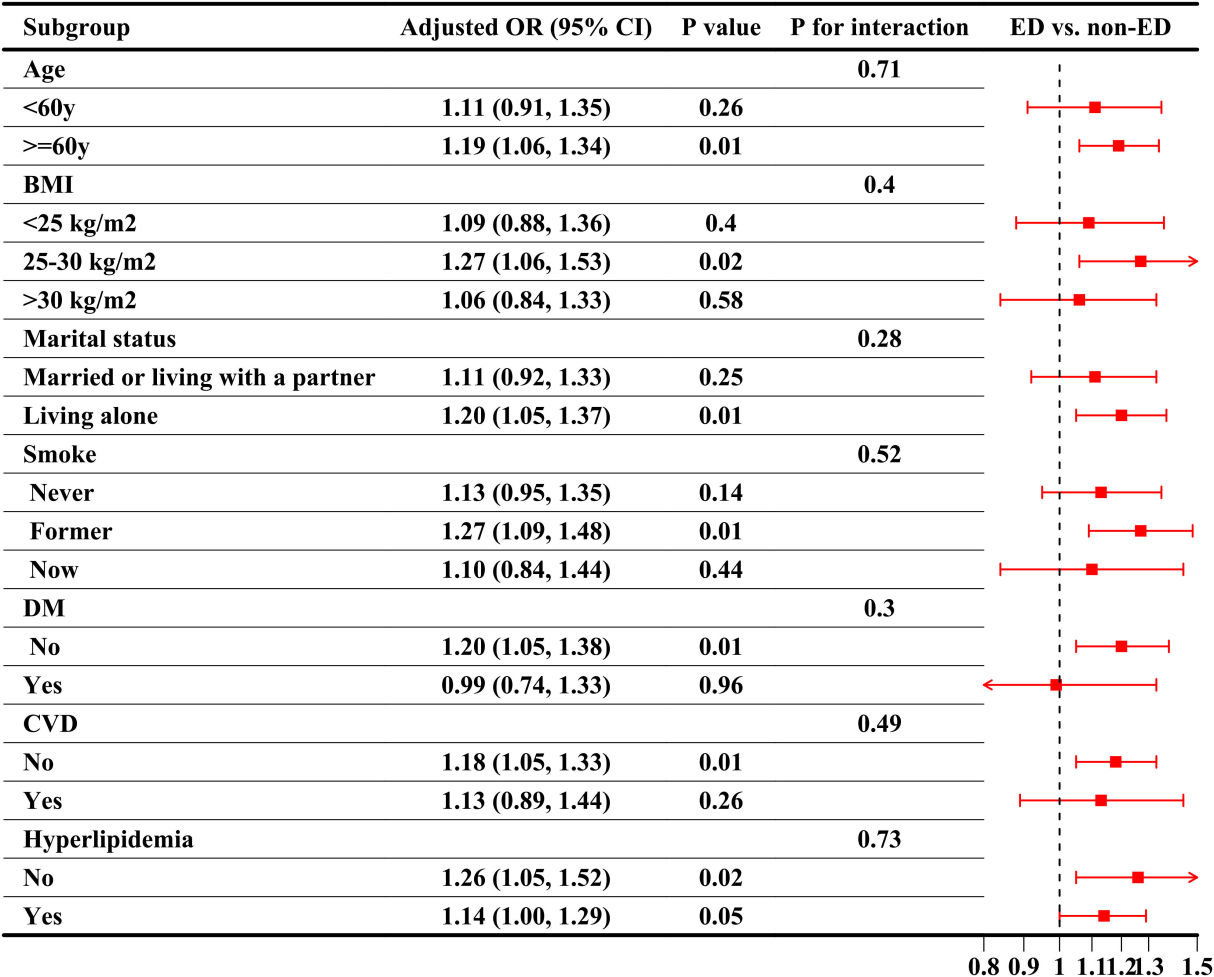
Subgroup analysis of the association between CRP/HDL ratio quartiles and severe ED, weighted. Subgroup analyses were conducted across various population characteristics, with CRP/HDL analyzed as quartiles (Q1 as the reference). All analyses were adjusted for covariates included in Model 3: age, race/ethnicity, education level, PIR, marital status, BMI, TC, UA, alcohol intake, smoking status, hypertension, DM, CVD, and hyperlipidemia, excluding the stratified variable itself. ORs with 95% CI are presented for each subgroup. Trend tests across quartiles and interaction tests to assess heterogeneity between subgroups were also conducted, with statistical significance defined as a two-sided p-value < 0.05. This sensitivity analysis focuses on severe ED, defined as participants who reported being “never able” to maintain an erection during sexual intercourse. CRP, C-reactive protein; HDL, High-density lipoprotein; BMI, Body mass index; PIR, Poverty income ratio; CVD, Cardiovascular disease; DM, Diabetes mellitus; ED, Erectile dysfunction; OR, Odds ratio; CI, Confidence interval.

In the sensitivity analysis, severe ED was defined as the outcome, and the previous analyses were repeated. First, **Table 4** presents the regression results, demonstrating that the association between CRP/HDL and severe ED remained statistically significant across all models. Specifically, in Model 3, the continuous CRP/HDL ratio was associated with an increased risk of severe ED, with an OR of 1.14 (95% CI: 1.03, 1.26; p = 0.02). When categorized into quartiles with Q1 as the reference, participants in Q3 and Q4 had significantly higher risks of severe ED, with ORs of 1.64 (95% CI: 1.17, 2.30; p = 0.01) and 1.45 (95% CI: 1.02, 2.06; p = 0.04), respectively. A trend test confirmed the significance of this dose-response relationship. The visualized trend in **Figure 4** also illustrated a clear linear pattern. Finally, **Table 5** and **Figure 5** present the stability of the relationship between continuous and quartile-categorized CRP/HDL and severe ED across different subgroups. The results indicate that the association remained consistent across subgroups, with no significant interactions detected, further validating the robustness of the CRP/HDL-ED relationship. These findings underscore the potential of CRP/HDL as a valuable marker for early detection of ED risk in clinical settings.

**Table 4:** Sensitivity analysis on the association between CRP/HDL ratio and severe ED, weighted.

Exposure	Model 1		Model 2		Model 3	
	OR (95%CI)	P value	OR (95%CI)	P value	OR (95%CI)	P value
Log (CRP/HDL*1000)	1.30(1.20,1.40)	<0.0001	1.19(1.09,1.29)	<0.001	1.14(1.03,1.26)	0.02
CRP/HDL*1000, quartiles						
Q1 (<1.556)	Reference		Reference		Reference	
Q2 (1.556-3.704)	1.51(1.16,1.96)	0.003	1.28(0.97,1.70)	0.08	1.21(0.85,1.73)	0.23
Q3 (3.704-8.444)	2.09(1.69,2.58)	<0.0001	1.76(1.32,2.34)	<0.001	1.64(1.17,2.30)	0.01
Q4 (>8.444)	2.32(1.77,3.03)	<0.0001	1.69(1.29,2.23)	<0.001	1.45(1.02,2.06)	0.04
P for trend	<0.0001		<0.001		0.015	

CRP, C-reactive protein; HDL, High-density lipoprotein; PIR, Poverty income ratio; BMI, Body mass index; TC, Total cholesterol; UA, Uric acid; CVD, Cardiovascular disease; ED, Erectile dysfunction; OR, Odds ratio; CI, Confidence interval.

The regression models evaluate the association between the CRP/HDL ratio and severe ED risk.

Model 1 includes only the CRP/HDL ratio without adjustment.

Model 2 adjusts for demographic factors (age, race/ethnicity, marital status, education level, and PIR).

Model 3 further adjusts for clinical factors (BMI, TC, UA, smoking status, alcohol consumption, hypertension, diabetes, CVD, and hyperlipidemia). The results are presented as ORs with 95% CIs, and P-values <0.05 are considered statistically significant. Trend tests across quartiles were conducted to assess the dose-response relationship. This sensitivity analysis redefines the outcome as severe ED, focusing on participants who reported being “never able” to maintain an erection during sexual intercourse.

**Table 5:** Subgroup analysis of the association between CRP/HDL ratio and severe ED across different population characteristics, weighted.

Subgroup	CRP/HDL*1000				P for trend	P for interaction
	Q1 (n=910)	Q2 (n=910)	Q3 (n=904)	Q4 (n=909)		
Age						0.12
<60y	Ref	1.11(0.71,1.74)	1.57(1.01,2.44)	1.14(0.70,1.86)	0.25	
≥60y	Ref	1.39(0.81,2.38)	1.65(1.12,2.45)	2.07(1.31,3.26)	0.003	
BMI						0.19
<25 kg/m^2^	Ref	1.49(0.89, 2.50)	1.81(1.12, 2.94)	1.17(0.60, 2.28)	0.10	
25-30 kg/m^2^	Ref	1.06(0.70,1.62)	1.72(1.03,2.87)	1.84(1.11,3.06)	0.01	
>30 kg/m^2^	Ref	0.98(0.43, 2.22)	1.05(0.51, 2.17)	0.88(0.44, 1.78)	0.65	
Marital status						0.99
Married or living with a partner	Ref	1.25(0.63,2.47)	1.71(1.01,2.89)	1.70(0.89,3.26)	0.05	
Living alone	Ref	1.23(0.81, 1.87)	1.62(1.06, 2.47)	1.37(0.89, 2.09)	0.05	
Smoking						0.36
Never	Ref	1.22(0.72, 2.06)	1.48(0.84, 2.61)	1.41(0.81, 2.45)	0.13	
Former	Ref	1.51(0.83, 2.74)	2.94(1.67, 5.19)	2.52(1.46, 4.33)	<0.001	
Current	Ref	1.12(0.68,1.83)	1.22(0.63,2.40)	1.05(0.53,2.06)	0.87	
DM						0.75
No	Ref	1.23(0.87,1.75)	1.71(1.22,2.39)	1.50(1.04,2.16)	0.01	
Yes	Ref	0.66(0.21, 2.02)	0.71(0.19, 2.67)	0.70(0.22, 2.19)	0.75	
CVD						0.35
No	Ref	1.16(0.83,1.61)	1.66(1.20,2.30)	1.46(1.00,2.15)	0.01	
Yes	Ref	2.21(0.67, 7.30)	1.59(0.49, 5.14)	1.38(0.38, 5.01)	0.98	
Hyperlipidemia						0.20
No	Ref	1.27(0.70, 2.32)	0.83(0.39, 1.75)	1.29(0.75, 2.22)	0.74	
Yes	Ref	1.24(0.77,1.98)	2.02(1.34,3.06)	1.58(1.04,2.40)	0.01	

CRP, C-reactive protein; HDL, High-density lipoprotein; BMI, Body mass index; PIR, Poverty income ratio; TC, Total cholesterol; UA, Uric acid; CVD, Cardiovascular disease; DM, Diabetes mellitus; ED, Erectile dysfunction; OR, Odds ratio; CI, Confidence interval.

All subgroup analyses were adjusted for the following covariates included in Model 3: age, race/ethnicity, education level, PIR, marital status, BMI, TC, UA, alcohol intake, smoking status, hypertension, DM, CVD, and hyperlipidemia, excluding the stratified variable itself. The CRP/HDL ratio was analyzed as quartiles (Q1 as the reference). Trend tests were conducted across quartiles, and interaction tests assessed heterogeneity between subgroups. Statistical significance was defined as a two-sided P-value < 0.05. This sensitivity analysis focuses on severe ED, defined as participants who reported being “never able” to maintain an erection during sexual intercourse.

## 4 Discussion

In this national population-based cross-sectional study, we are the first to identify a significant positive association between the CRP/HDL ratio and ED. Our results demonstrate that elevated levels of this novel inflammation-lipid composite marker are consistently linked with an increased risk of ED across different models and subgroups. Notably, this association remained robust even after adjusting for a wide range of potential confounders. Sensitivity analyses further reinforced these findings, suggesting that the CRP/HDL ratio could serve as a valuable marker for predicting ED risk and facilitating early clinical interventions.

The CRP/HDL ratio combines the predictive values of CRP and HDL into a single novel marker of inflammation and lipid metabolism, potentially reflecting the dynamic interplay between various pro-inflammatory and anti-inflammatory mediators (28). Compared to individual markers like CRP or HDL alone, the CRP/HDL ratio offers greater predictive advantages in relation to certain diseases. Current studies have already demonstrated that the combination of low HDL-C and elevated CRP levels is associated with adverse outcomes in patients with ischemic stroke, heart failure, and coronary artery disease (35–37). In line with these findings, our study establishes a significant correlation between the CRP/HDL ratio and the incidence of ED, suggesting that this composite marker could serve as a valuable indicator for assessing the risk of developing ED.

ED is closely associated with CVD, with endothelial dysfunction being a fundamental pathophysiological mechanism linking the two conditions (38, 39). Under normal circumstances, the vascular endothelium has the capacity to regulate hemostasis, inflammation, and repair processes in response to local injury, while maintaining steady blood flow to prevent inflammation (14, 40). However, when the balance within the body is disrupted, increased oxidative stress and elevated levels of inflammation can lead to endothelial dysfunction (41). This dysfunction results in the exposure of endothelial cells to pro-inflammatory cytokines, thereby inhibiting vasodilation and ultimately contributing to the development of ED (42, 43). Consequently, inflammation emerges as a key mediator of endothelial dysfunction, and CRP, a well-established marker of inflammation, has been shown to be closely associated with ED in numerous studies (14, 18).

In addition to its relationship with CRP, HDL is also intimately connected to endothelial function. HDL possesses several crucial biological properties, including cholesterol reverse transport, antioxidant, anti-inflammatory, endothelial/vasodilatory, antithrombotic, and cytoprotective effects (44). HDL-C mitigates the oxidative effects of low-density lipoprotein cholesterol (LDL-C), thereby inhibiting the pro-inflammatory activation of endothelial cells triggered by LDL-C (45). Moreover, HDL is capable of reducing the expression of adhesion molecules on the endothelial cell surface, such as P-selectin, E-selectin, intercellular adhesion molecule-1 (ICAM-1), and vascular cell adhesion molecule-1 (VCAM-1), thereby counteracting inflammation (46). By reducing the expression of these proteins, HDL prevents the adhesion and migration of T lymphocytes and monocytes to the vascular endothelium and their subsequent infiltration into atherosclerotic areas (47, 48). Additionally, HDL has been shown to increase the expression of endothelial nitric oxide synthase (eNOS), which plays a critical role in vasodilation by promoting the production of nitric oxide (NO) (49). This function is essential for maintaining normal blood flow and erectile function. Therefore, a decrease in HDL-C is strongly associated with endothelial dysfunction and the pathogenesis of ED.

In summary, CRP and HDL are crucial markers of inflammation and endothelial dysfunction, respectively, each playing significant roles in the promotion and inhibition of inflammatory responses. Their involvement in the pathophysiology of ED underscores the potential of the CRP/HDL ratio as a valuable indicator for assessing the risk of ED. However, several limitations of this ratio must be acknowledged. Firstly, CRP reflects not only chronic inflammation but also acute inflammation when levels exceed 10 mg/L (50). Therefore, to accurately assess ED risk, it is essential to exclude acute inflammatory phases, as transient CRP elevations could confound the interpretation. Secondly, although HDL elevation generally exerts protective vascular effects, its benefits do not increase indefinitely. Excessively high HDL levels may exert opposite effects, potentially damaging the vasculature and increasing the risk of CVD (51)​. Lastly, HDL-C primarily reflects the quantity of HDL rather than its functionality, which includes anti-inflammatory, antioxidant, and cholesterol efflux properties—all of which contribute to vascular health (52). While HDL-C is a convenient clinical biomarker, it has limitations as a surrogate for HDL functionality. Therefore, future clinical use of the CRP/HDL ratio should define specific reference ranges, and direct measures of HDL functionality should replace HDL-C to provide a more accurate assessment.

The large sample size and detailed statistical methods in our study are commendable strengths, but potential limitations must still be recognized and addressed. First, as with all cross-sectional studies, causality cannot be inferred from our findings. Second, our analysis is based on data from the 2001-2004 NHANES cycles, and demographic characteristics have since changed significantly, limiting the generalizability of our results to current populations. Third, while the CRP/HDL ratio shows promise as a predictive marker, the simplicity and subjectivity of the single-question assessment used to evaluate ED may have introduced bias. Moreover, the reliance on outdated data underscores the need for further validation with more recent datasets. Finally, while our findings suggest the utility of CRP/HDL in early ED detection and intervention, additional large-scale longitudinal studies are needed to confirm its predictive value across diverse populations. Future research should also employ more comprehensive methods, such as color Doppler duplex ultrasound (CDDU), to assess penile arterial function and delve deeper into the underlying mechanisms linking CRP/HDL with vascular damage.

## 5 Conclusion

Our study, leveraging the nationally representative NHANES dataset, confirms the association between a novel inflammation-lipid composite marker, CRP/HDL, and ED. This marker integrates both inflammatory and lipid metabolic pathways, which are critical mechanisms underlying the pathogenesis of ED. The strong correlation between CRP/HDL and ED suggests a promising avenue for early ED screening and risk assessment. Incorporating this marker into clinical practice could facilitate timely identification of ED risk and enable early interventions, ultimately reducing the disease burden. However, the limitations of this study highlight the need for future large-scale cohort studies to validate our findings and further investigate the underlying pathological mechanisms.

## Data Availability

The original contributions presented in the study are included in the article/supplementary material. Further inquiries can be directed to the corresponding authors.
